# Foot orthoses alter lower limb biomechanics but not jump performance in basketball players with and without flat feet

**DOI:** 10.1186/s13047-019-0334-1

**Published:** 2019-04-23

**Authors:** Malia Ho, Pui Wah Kong, Lowell Jia-Yee Chong, Wing-Kai Lam

**Affiliations:** 1 Department of Podiatry, School of Health, Medical and Applied Science, Central Queensland University, Rockhampton, Queensland Australia; 2 Physical Education and Sports Science Academic Group, National Institute of Education, Nanyang Technological University, Singapore, Singapore; 3 Department of Kinesiology, Shenyang Sport University, Shenyang, China; 4 Li Ning Sports Science Research Center, Beijing, China

**Keywords:** Basketball, Jump take-off, Joint moment, Jump push-off, Countermovement jump, Standing broad jump

## Abstract

**Background:**

Flat-footed individuals are believed to have poorer jump performance compared to normal-arched individuals. Foot orthoses are commonly used to support the deformed foot arch, and improve normal foot function. However, it is unclear if foot orthoses use affects jump performance in athletes. Our study aims to investigate if foot type and/or foot orthosis influence countermovement jump (CMJ) and standing broad jump (SBJ) performance and lower limb biomechanics.

**Methods:**

Twenty-six male basketball players were classified into normal-arched (*n* = 15) or flat-footed (*n* = 11) groups using the Chippaux-Smirak index, navicular drop test, and the resting calcaneal angle measurement. They performed jumps with and without prefabricated foot orthoses. We measured jump height and distance for CMJ and SBJ, respectively. Hip, knee and ankle joint angles, angular velocities, moments and powers during take-off were also measured.

**Results:**

For CMJ, the flat-footed group exhibited less ankle plantarflexion (*F*_1,24_ = 8.407, *p* = 0.008, *η*_p_^2^ = 0.259 large effect) and less hip joint power (*F*_1,24_ = 7.416, *p* = 0.012, *η*_p_^2^ = 0.244 large effect) than the normal-arched group. Foot orthoses reduced ankle eversion in both groups (*F*_1,24_ = 6.702, *p* = 0.016, *η*_p_^2^ = 0.218 large effect). For SBJ, the flat-footed group produced lower peak hip angular velocity (*F*_1,24_ = 7.115, *p* = 0.013, *η*_p_^2^ = 0.229 large effect) and generated lower horizontal GRF (*F*_1,24_ = 5.594, *p* = 0.026, *η*_p_^2^ = 0.189 large effect) than the normal-arched group. Wearing foot orthoses reduced ankle eversion (*F*_1,24_ = 5.453, *p* = 0.028, *η*_p_^2^ = 0.185 large effect), peak horizontal GRF (*F*_1,24_ = 13.672, *p* = 0.001, *η*_p_^2^ = 0.363 large effect) and frontal plane ankle moment (*F*_1,24_ = 4.932, *p* = 0.036, *η*_p_^2^ = 0.170 large effect).

**Conclusion:**

Foot type and the use of foot orthoses influence take-off biomechanics, but not actual CMJ and SBJ performances in basketball players. Compared to the normal-arched individuals, flat-footed athletes generated smaller propulsion GRF and lower hip flexion velocity and power, which suggests possible compensatory movement strategies to maximise jump performance. Future studies may investigate whether these altered biomechanics, taking into consideration their respective magnitude and effect sizes, may have implications on lower limb injuries. The use of foot orthoses resulted in biomechanical changes in both the normal-arched and flat-footed groups but does not enhance jumping performance.

## Background

Jumping is one of the most common manoeuvres performed by basketball players in a game. For example, in a competitive basketball game, each player performs 44 jumps on average [1]. Individuals with flat feet in particular have been found to demonstrate poor ability to control foot movements in the ankle and foot complex [2], which may lead to poor jump performance. However, the association between flat feet and jumping performance has not been fully investigated.

One study found no significant relationship between the foot arch height and the vertical jump height in both normal-arched and flat-footed individuals [3]. Other studies showed that having flat feet did not affect the motor performances in vertical jumps, sprints and static balance in children [4]. The association between arch height and sporting ability agrees with recent findings that static foot posture measurements poorly predicts how the foot will function dynamically [5].

While these studies compared jumps performed in barefoot conditions [3–5], it is unclear how jump performance may be affected in shod conditions. In basketball, players use footwear in all training and competition. One study found that the use of footwear has the capacity to influence the jump performance of athletes [6]. Studies measuring jump performances barefoot may therefore not reflect accurately jump performances during games where footwear is used regularly. Therefore, an investigation into jump performance with the participants wearing footwear would provide a more realistic reflection of jump performance during a game.

Foot orthoses were designed for individuals with flat feet, to provide support for the medial longitudinal foot arch, restoring normal lower limb movement patterns [7]. Foot orthoses have been reported to successfully reduce the magnitude of foot and leg movements and produce shock attenuation [8]. Foot orthoses have also been found to reduce postural sway and energy consumption during functional tasks [9]. While the use of orthoses has been reported to also successfully reduce painful foot symptoms in athletes [10], there is a lack of evidence supporting the use of foot orthoses to improve jumping performance for athletes. One study showed that flat-footed athletes demonstrated different lower limb biomechanics with and without using foot orthoses. As actual jump performance was not reported [11] in this study, inference on performance enhancement could not be drawn. It is necessary to further examine the effects of foot type and foot orthoses use on jumping outcomes and biomechanics in athletes.

Our study aimed to investigate the effects of foot type and foot orthoses on the jump performance and lower limb biomechanics of trained basketball players. We hypothesised that 1) flat-footed basketball players would exhibit poorer jumping performance when compared to normal-arched basketball players, and 2) the jump performance and take-off biomechanics of normal and flat footed basketball players is improved when wearing foot orthoses compared to when not wearing foot orthoses. The results from our study would provide evidence of whether using foot orthoses would be beneficial for flat-footed basketball players performing jump tasks.

## Methods

### Participant

Twenty-six male basketball players were recruited from the basketball teams in three local universities in Beijing, China. Written informed consent was obtained from all participants before data collection and ethical approval was granted from institutional ethics committee (IRB2017BM005). Basketball players were chosen because they were familiar with the technique and execution of a variety of jumps in basketball games [12]. Each participant’s foot length was measured using the Brannock foot measurement device (Brannock Device, Syracuse, NY, USA). Only participants with a foot length within the range of US size 8.5 to 11.5 for both feet were recruited as these were the available sizes (9, 10, 11) of footwear and prefabricated foot orthoses used in our study. All participants were free of any lower extremity injuries for at least 6 months prior to the start of the study. Participants who had current or previous foot orthotic therapy/intervention were excluded from the study to prevent any bias due to previous exposure to orthoses.

Participants were included if they had normal-arched feet or flat feet. While there are many different ways of foot type classification, there is currently no consensus on which method is the most appropriate [13]. To improve the validity and reliability of foot type classification in our study, we chose to classify a foot as flat footed only if the outcome measures of at least two out of three screening tests agreed. The three tests chosen were: The Chippaux Smirak Index [14], navicular drop test [15] and the resting calcaneal stance position measurement [16]. These tests were chosen as they were individually found to correlate to radiographic measures of the skeletal structure of the foot [17–19]. Participants were deemed eligible if both their left and right feet were classified as either normal-arched or flat-footed based on the criteria set out in two of the three screening tests.

#### Chippaux-Smirak index

The participants had to walk over a pedograph to obtain an inked footprint. The Chippaux-Smirak index was measured manually and determined as the ratio of the widest part of the forefoot to the narrowest part of the foot arch measured from the footprint. The index is expressed as a percentage and a score of 45.05% and above would classify a foot arch as flat; a foot with a score less than 45.05% classifies a foot arch as normal [13]. The Chippaux-Smirak index has been found to be accurate in predicting flat foot with a sensitivity of 94.2% [14].

#### Navicular drop test

The navicular drop test is used to measure the degree of arch collapse in order to classify foot types [15]. The navicular tuberosity is palpated on the foot and marked. The weight bearing foot is placed in the neutral calcaneal stance position (NSCP) and the vertical distance of the navicular tuberosity to the ground is measured. The foot is then allowed to relax and settle in the relaxed calcaneal stance position. The vertical distance of the navicular tuberosity to the ground was measured again and the difference between the two measurements was calculated. A flat foot is defined as a foot that demonstrated a navicular drop of more than 1.0 cm. A normal-arched foot exhibits equal or less than 1.0 cm of navicular drop. The measurement of the navicular drop was shown to exhibit high levels of intra-rater reliability but poor to moderate levels of inter-rater reliability [20]. The ability to correctly locate the navicular tuberosity landmark as well as the ability to correctly place the foot in NCSP relies on the experience and competency of the assessor [21]. In our study, only one assessor conducted all measurements to minimise the measurement errors due to multiple testers.

#### Resting calcaneal stance angle measurement

For the resting calcaneal stance angle measurement, the degree of angular deviation of the calcaneal bisection line from perpendicular is measured using a goniometer [16]. A flat foot is one with a calcaneal angle of more than 5 degrees, while a normal-arched foot has a calcaneal angle of between 0 and 5 degrees [16]. This method is a common method used by clinicians to classify foot structure in a clinical setting as the calcaneal bisection line can be quickly drawn and the angle measured quickly.

Based on the results of the three tests, we classified our participants into normal-arched group [*n* = 15, mean ± SD: age 21.47 ± 4.10 years, height 1.80 ± 0.03 m, mass 74.09 ± 10.95 kg, years of playing experience 7.20 ± 4.39 years, Chippaux Smirak Index 34.09 ± 6.59%, Navicular Drop 5.10 ± 2.22 mm, Resting Calcaneal Stance Ankle 3.03 ± 2.70°] and flat-footed group [*n* = 11, mean ± SD: age 23.00 ± 4.67 years, height 1.78 ± 0.05 m, mass 76.38 ± 10.42 kg, years of playing experience 9.10 ± 4.14 years, Chippaux Smirak Index 45.51 ± 6.75%; Navicular Drop 9.90 ± 2.22 mm, Resting Calcaneal Stance Ankle 5.95 ± 1.88°]. There were significant differences between normal-arched and flat-footed groups in all foot screening measurements (*p* < 0.050).

### Foot orthoses conditions

**Fig. 1:**
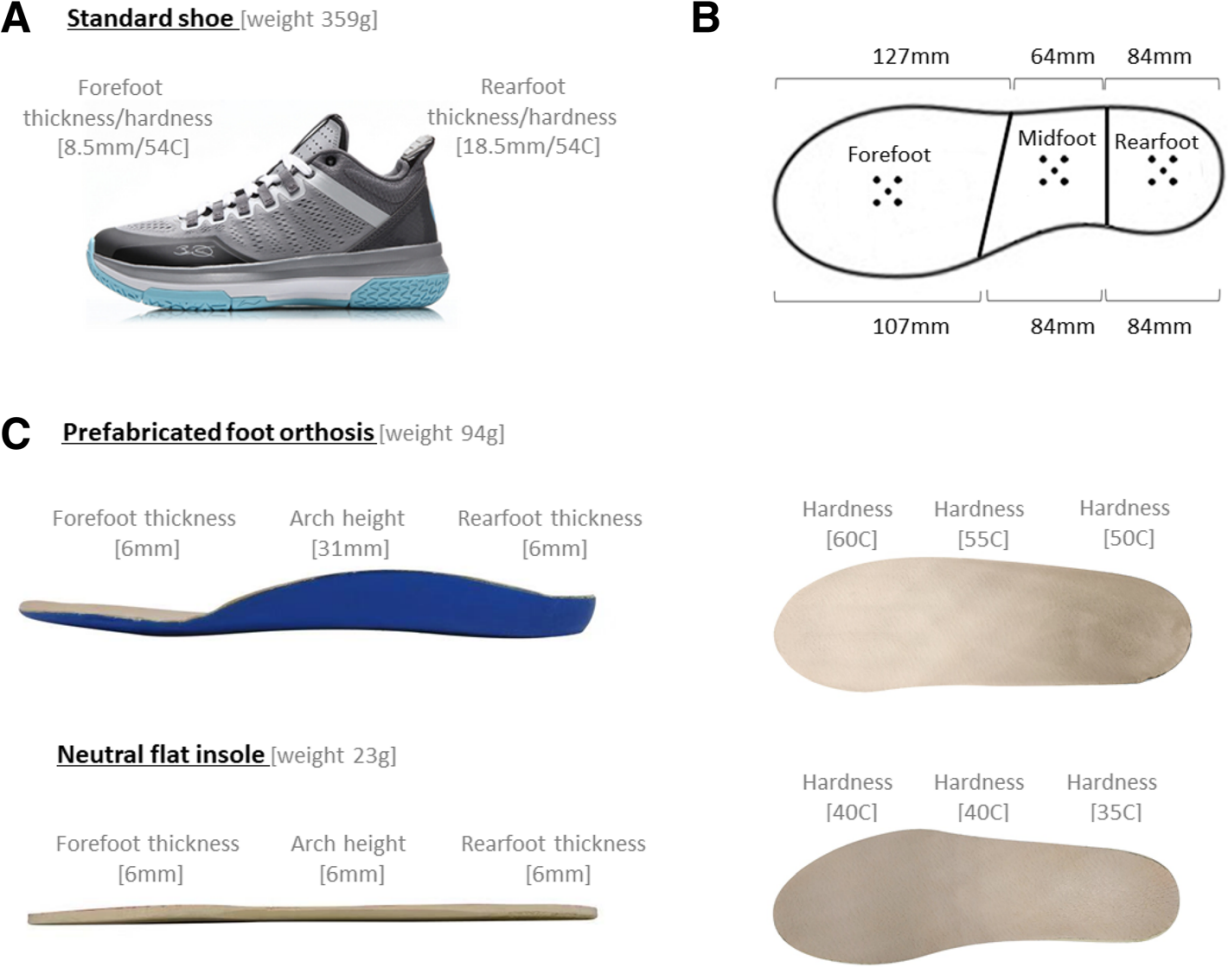
Dimensions and properties of US9 **a**: Standard shoe, **b**: Foot regions, and **c**: Foot orthoses

Our participants performed their jump trials under 2 foot orthoses conditions. Standard basketball shoes (Wade All Day 2, ABPM013, Beijing, China) (Fig. 1 a) in US sizes of 9.0, 10.0 or 11.0 were provided to each participant. For the experimental condition, commercially available prefabricated foot orthoses (Firm Orthotic Insole, Salford Insole, UK) were used as it has been clinically validated to reduce pronation in the foot [22] Furthermore, the material is reported to be durable and waterproof, suitable to meet the stresses of high intensity sports and to meet foot hygiene requirements for athletes [22]. For the control condition, the neutral flat insoles with minimal arch support was used. We measured the dimensions and hardness of the insoles used in sections as illustrated in Fig. 1b and the specifications of the insoles is shown in Fig. 1c. To blind the participants to the type of insoles that were used, we covered all insoles with a thin layer of leather.

### Jump tasks

We used the vertical countermovement jump (CMJ) and standing broad jump (SBJ) for this study as these jump tasks are commonly used to assess the explosive strength of the lower body. Good performance in CMJ and SBJ have also been correlated to sports proficiency [23].

#### Countermovement jump

Before trial acquisition, we recorded the standing reach height of participants as a baseline. The participant started by standing upright on the force platform and then performed the CMJ by going into a squatted position with hips and knees flexed, before extending the legs to jump up vertically and off the ground with their maximum-effort. All participants were instructed to swing their arms in the CMJ and use their right hand to touch the vanes of the Vertec measuring equipment (Vertec, Sports Imports, Hilliard, OH, USA) at the highest point of their jump. A successful jump required the participant to perform a double-leg take-off and land within the force platform, while maintaining balance after landing without stepping out of the force platform. We recorded five successful trials of CMJ for each insole condition.

#### Standing broad jump

We asked all participants to stand upright with the edge of the force platform. The participant then performed the SBJ with maximum-effort, by bending the hips and knees and going to a squatted position together with the arm swing, followed by the extension of the legs and forward lean of the body for take-off. A successful jump required the participant to perform a double-leg take-off and land with both feet, while maintaining balance after landing. Five successful trials of SBJ were recorded for each insole condition.

### Procedures

We placed 22 retro-reflective markers on various anatomical landmarks of the participants [24, 25]. These anatomical landmarks included: right and left anterior superior iliac spine, right and left posterior superior iliac spine, lateral and medial femoral epicondyles, medial and lateral malleolus, medial side of the first metatarsal head, lateral side of the fifth metatarsal head, posterior upper, posterior lower and lateral aspect of the calcaneus. We also attached two four-marker rigid clusters onto the thigh and shank segments, respectively.

To standardise the foot-shoe interface, a new pair of standardised socks was given to participants. We asked participants to tighten their laces to their individual preference. The participants had 2 minutes of self-directed warm-up to familiarise themselves with the given pair of insoles. Participants had three familiarisation jump trials before the actual data collection of CMJ and SBJ trials. The orders of insole and jump task conditions were randomly presented to the participants. We used a 90 × 60 cm force plate (Advanced Mechanical Technology Inc., Watertown, MA, USA) to collect the take-off ground reaction force (GRF) of the jump tasks at a sampling rate of 1000 Hz. Ten synchronised VICON-T040 infrared cameras (Vicon, Metrics Ltd., Oxford, UK) were used to record joint kinematic data, with a capturing frequency of 200 Hz.

### Data processing

**Fig. 2:**
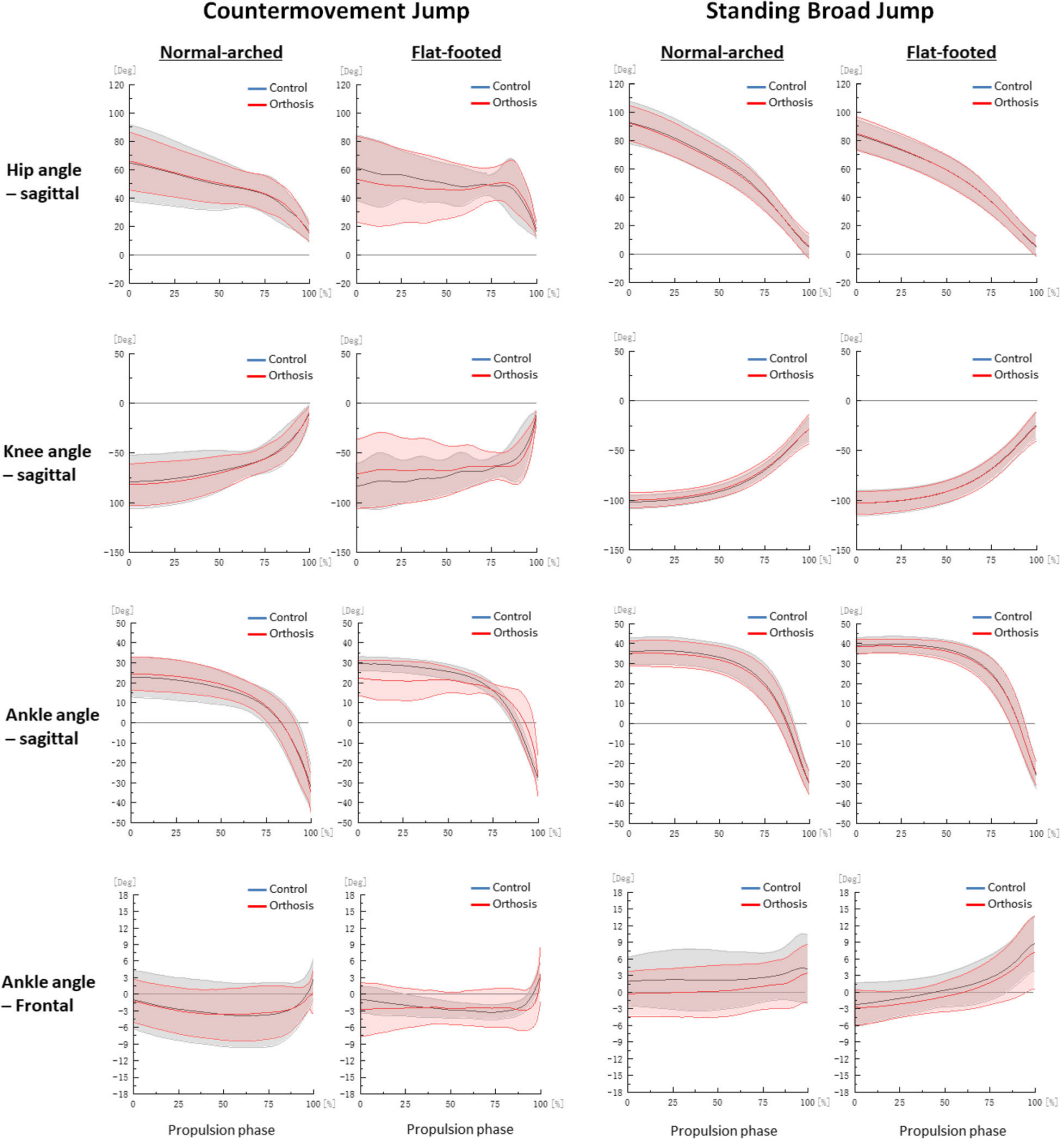
Hip, knee and ankle angle curves during propulsion phase by foot type and orthosis

**Fig. 3:**
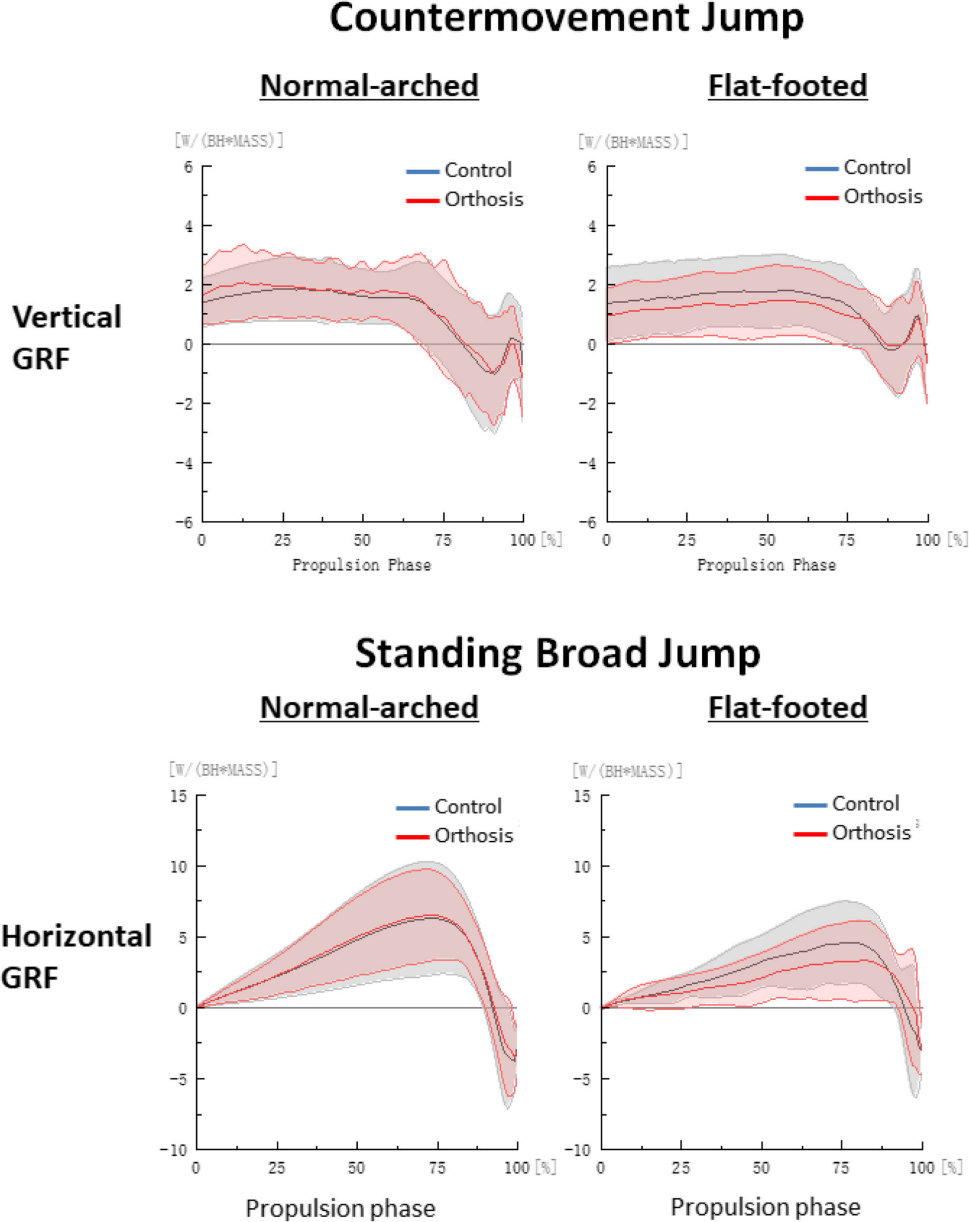
Vertical and horizontal curves during propulsion phase by foot type and orthosis

**Fig. 4:**
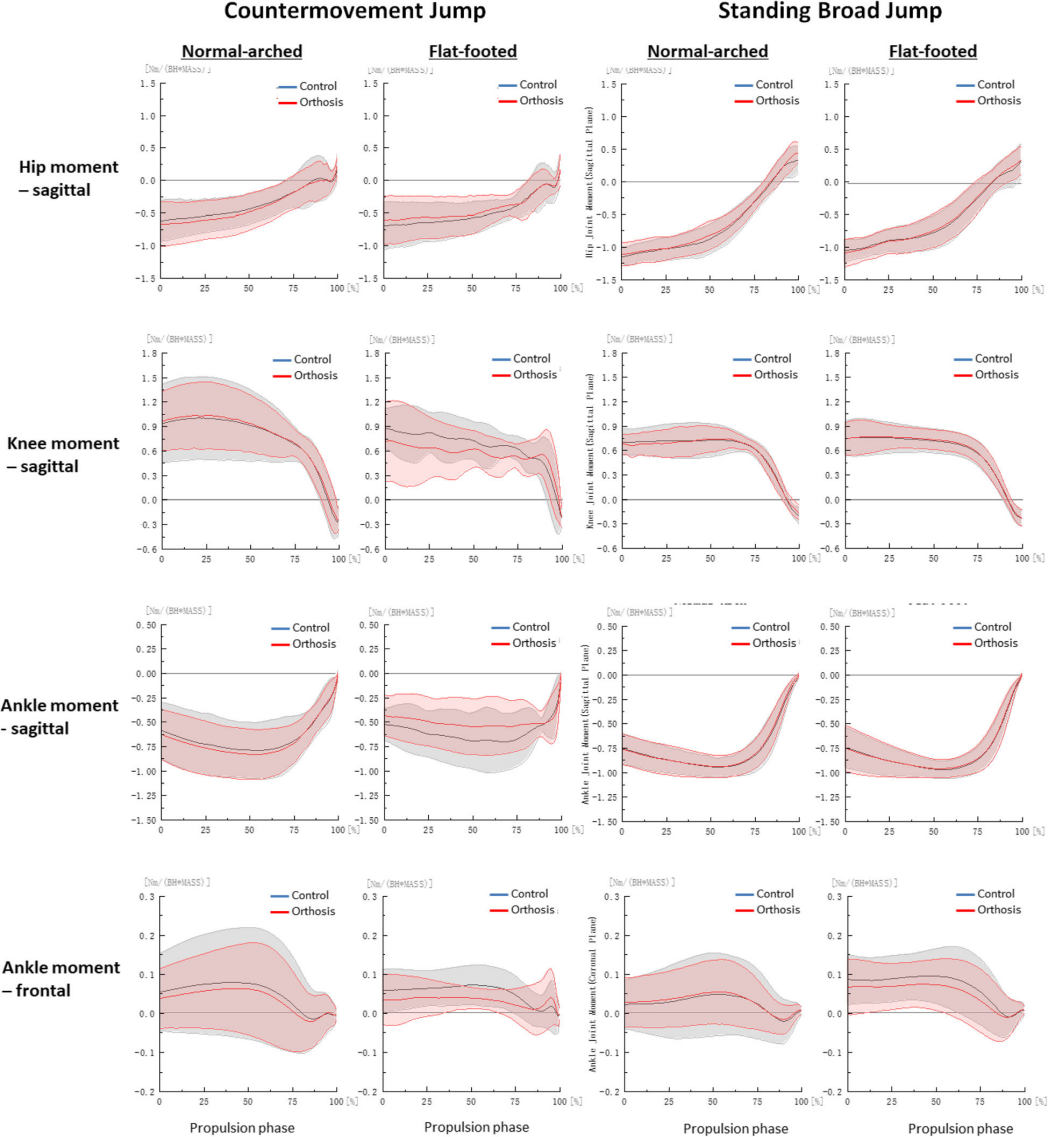
Hip, knee and ankle moment curves during propulsion phase by foot type and orthosis

**Fig. 5:**
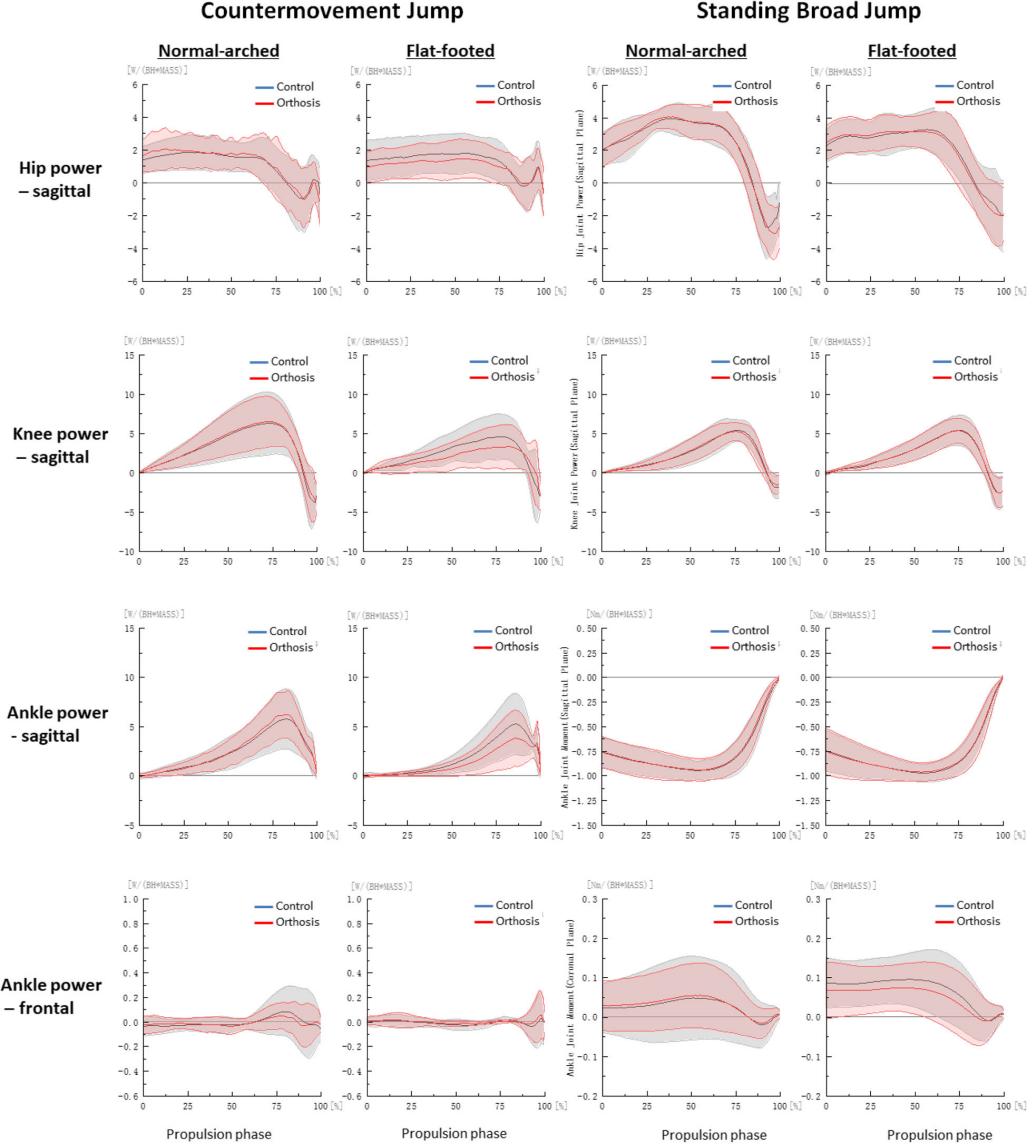
Hip, knee and ankle power curves during propulsion phase by foot type and orthosis

We derived jump height by subtracting the dynamic jump height from the standing reach height of the right arm obtained from the Vertec, and we measured the jump distance from the start line to the back of the heel of the participant after landing [26]. All marker trajectories were identified manually using Vicon Clinical Manager Software (Oxford Metrics Ltd., Oxford, UK). A spline interpolation was performed for minor missing data using three frames of data before and after the missing data (Fig. 2). Kinematic and GRF data were filtered with a fourth order Butterworth low-pass filter with a cut-off frequency of 13.33 Hz [27]. The biomechanical variables measured for both jumps were hip, knee and ankle (sagittal and frontal) take-off angles, peak angular velocities, peak sagittal joint moments and powers were calculated as these variables are of direct relevance to the evaluation of athletic performances and foot orthosis [2, 8, 11, 24, 25, 28]. The instant of take-off was defined as when the vertical GRF first falls below 3.0 N [29]. Braking and propulsion phases were determined by the instant of maximum knee flexion [30]. Joint angle was defined as the orientation of the distal segment relative to the proximal segment and joint moment was defined through an inverse dynamic model in Visual 3D (C-Motion Inc., Ontario, Canada) [24, 25, 30]. Joint power was defined as the dot product of the joint moment and angular velocity. A positive value for joint angle, moment and power denoted flexion, eversion and internal rotation for respective orthogonal planes, with zero degree defined at neutral standing position. All kinetic data was normalised to body weights [24, 25, 30] (Figs. 2, 3, 4 and 5).

### Statistical analysis

We performed statistical analyses using SPSS (version 24; IBM Corp., Armonk, NY). For each of the two jump tasks, a 2 × 2 (Foot type × Orthosis) mixed analysis of variance (ANOVA) was conducted on jump performance, kinematic and kinetic variables. The effect size (partial Eta-squared, *η*_p_^2^) were calculated and interpreted as small (0.1 < *η*_p_^2^ < 0.06), medium (0.06 ≤ *η*_p_^2^ < 0.14) and large (*η*_p_^2^ ≥ 0.14) [31]. Level of significance was set at *p* < 0.05. Data was expressed as mean ± SD.

## Results

### Countermovement jump

There were no significant interactions between foot type and foot orthoses for all jump height, kinematics and kinetic variables (*p* > 0.05, Tables 1 and 2). There were also no effects of foot type (*F*_1,24_ = 0.712, *p* = 0.407, *η*_p_^2^ = 0.029 small effect) and foot orthosis (*F*_1,24_ = 3.248, *p* = 0.084, *η*_p_^2^ = 0.119 medium effect) on vertical jump height.

**Table 1:** Countermovement jump kinematics of participants when wearing control and prefabricated orthosis

Variables	Foot type group	Control	Orthosis	MD_orthosis_ [95% CI]	ANOVA *p*-value		
		(Mean ± SD)	(Mean ± SD)		Foot type	Orthosis	Interaction
Joint angles at take-off							
Hip - sagittal (°)	Normal arched	15.5 ± 6.4	14.4 ± 5.6	0.9 [− 0.4,2.1]	.911	.393	.323
	Flat-footed	15.3 ± 4.0	15.3 ± 5.0	− 0.1 [− 1.9,2.3]			
	MD_foot_ [95% CI]	0.2 [−4.3,4.8]	−0.7 [−5.1,3.7]				
Knee - sagittal (°)	Normal arched	8.1 ± 4.8	8.0 ± 3.7	− 0.1 [− 1.2,1.1]	.144	.919	.848
	Flat-footed	10.3 ± 4.2	10.4 ± 3.1	1.10 [−0.8,3.0]			
	MD_foot_ [95% CI]	2.2 [−1.5,5.9]	2.4 [−0.4,5.2]				
Ankle - sagittal (°)	Normal arched	36.2 ± 3.6	37.0 ± 9.0	0.8 [−3.0,4.6]	**.008**	.972	.506
	Flat-footed	29.9 ± 6.6	29.2 ± 6.3	−0.7 [− 2.3,0.9]			
	MD_foot_ [95% CI]	−6.3 [−10.5,-2.1]	− 7.8 [− 14.3,-1.2]				
Ankle - frontal (°)	Normal arched	3.4 ± 4.2	0.7 ± 4.3	2.7 [0.4,5.0]	.186	**.016**	.287
	Flat-footed	4.8 ± 3.8	3.7 ± 5.2	1.10 [− 0.8,3.0]			
	MD_foot_ [95% CI]	−1.4 [− 4.5,2.0]	−3.0 [−6.8,0.9]				
Peak joint angular velocity							
Hip - sagittal (°/s)	Normal arched	522.1 ± 175.8	531.9 ± 135.3	9.8 [−29.8,49.4]	.493	.250	.110
	Flat-footed	519.3 ± 98.7	461.4 ± 128.3	−58.0 [−146.6,30.8]			
	MD_foot_ [95% CI]	−2.8 [− 125.6120.0]	−70.5 [− 180.8,39.8]				
Knee - sagittal (°/s)	Normal arched	926.8 ± 227.3	977.5 ± 130.9	− 51.7 [− 131.5,28.1]	.976	.850	.107
	Flat-footed	982.2 ± 58.5	917.2 ± 239.2	65.0 [−75.4205.4]			
	MD_foot_ [95% CI]	−56.4 [− 202.4,89.6]	60.3 [−94.6215.3]				
Ankle - sagittal (°/s)	Normal arched	892.2 ± 229.8	1147.0 ± 731.6	254.8 [− 137.5647.0]	.251	.407	.131
	Flat-footed	901.1 ± 79.7	825.1 ± 216.1	−76.0 [−198.2,46.1]			
	MD_foot_ [95% CI]	8.8 [− 141.7159.4]	− 321.9 [− 795.5151.6]				
Ankle - frontal (°/s)	Normal arched	329.3 ± 158.5	296.5 ± 151.5	32.8 [−8.8,74.5]	.957	.053	.992
	Flat-footed	332.4 ± 119.2	299.9 ± 171.3	32.5 [−26.8,91.8]			
	MD_foot_ [95% CI]	−3.1 [− 122.0,115.9]	−3.4 [− 137.1130.3]				

MD_foot_ = mean difference in foot type (flat foot – normal arch), MD_foot_ = mean difference in orthosis condition (orthosis – control), *CI* confidence intervals, *ANOVA* Analysis of Variance.*Significant differences (*p* < .05) are shown in bold

**Table 2:** Countermovement jump kinetics of participants when wearing control and prefabricated orthosis

Variables	Foot type group	Control	Orthosis	MD_orthosis_ [95% CI]	ANOVA *p*-value		
		(Mean ± SD)	(Mean ± SD)		Foot type	Orthosis	Interaction
Peak vertical GRF (%BW)	Normal arched	1.39 ± 0.20	1.38 ± 0.18	0.01 [− 0.01,-0.03]	.059	.332	.808
	Flat-footed	1.26 ± 0.09	1.26 ± 0.09	0.01 [− 0.01,0.03]			
	MD_foot_ [95% CI]	0.12 [0.01,0.26]	0.12 [0.00,0.25]				
Peak joint moment							
Hip - sagittal (Nm/kg)	Normal arched	0.51 ± 0.56	0.47 ± 0.48	0.04 [0.40,0.48]	.071	.896	.827
	Flat-footed	0.27 ± 0.16	0.27 ± 0.15	−0.01 [− 0.05,0.04]			
	MD_foot_ [95% CI]	0.25 [− 0.11,0.60]	0.19 [− 0.12,0.51]				
Knee - sagittal (Nm/kg)	Normal arched	1.22 ± 0.35	1.28 ± 0.23	− 0.06 [− 0.15,0.04]	.495	.968	.188
	Flat-footed	1.20 ± 0.27	1.14 ± 0.33	−0.05 [− 0.10,0.20]			
	MD_foot_ [95% CI]	0.03 [−0.24,0.29]	0.13 [−0.10,0.36]				
Ankle - sagittal (Nm/kg)	Normal arched	0.97 ± 0.21	1.01 ± 0.13	0.04 [−0.02,0.09]	.219	.774	.086
	Flat-footed	0.94 ± 0.12	0.88 ± 0.18	−0.05 [− 0.15,0.05]			
	MD_foot_ [95% CI]	−0.04 [− 0.18,0.11]	−0.12 [− 0.25,0.01]				
Ankle - frontal (Nm/kg)	Normal arched	0.13 ± 0.11	0.12 ± 0.09	0.01 [− 0.01,0.03]	.910	.122	.980
	Flat-footed	0.13 ± 0.06	0.12 ± 0.07	0.01 [− 0.01,0.03]			
	MD_foot_ [95% CI]	0.00 [−0.07,0.07]	0.00 [−0.06,0.07]				
Peak joint power							
Hip - sagittal (W/kg)	Normal arched	4.18 ± 3.96	5.36 ± 6.97	−1.18 [−2.97,0.61]	**.012**	.217	.225
	Flat-footed	0.27 ± 0.16	0.27 ± 0.15	−0.01 [− 0.05,0.03]			
	MD_foot_ [95% CI]	3.91 [1.43,6.39]	5.08 [0.71,9.45]				
Knee - sagittal (W/kg)	Normal arched	8.14 ± 2.93	8.33 ± 1.82	− 0.18 [−1.15,0.79]	.122	.716	.392
	Flat-footed	7.22 ± 1.41	6.77 ± 1.77	0.45 [− 0.87,1.78]			
	MD_foot_ [95% CI]	0.92 [−1.07,2.91]	1.56 [0.05,3.06]				
Ankle - sagittal (W/kg)	Normal arched	7.31 ± 2.54	7.65 ± 1.78	−0.34 [−1.04,0.37]	.829	.594	.112
	Flat-footed	7.66 ± 1.01	6.98 ± 2.12	0.67 [−0.56,1.90]			
	MD_foot_ [95% CI]	−0.34 [−1.91,1.21]	0.67 [−0.95,2.28]				
Ankle - frontal (W/kg)	Normal arched	0.18 ± 0.23	0.16 ± 0.12	0.02 [− 0.06,0.10]	.803	.693	.588
	Flat-footed	0.15 ± 0.17	0.15 ± 0.20	0.00 [−0.05,0.05]			
	MD_foot_ [95% CI]	0.03 [−0.14,0.20]	0.01 [− 0.13,0.14]				

*GRF* ground reaction force, *MD*_*foot*_ mean difference in foot type (flat foot – normal arch), *MD*_*foot*_ mean difference in orthosis condition (orthosis – control), *CI* confidence intervals, *ANOVA* Analysis of Variance. *Significant differences (*p* < .05) are shown in bold

The main effect of foot type indicated that the flat-footed group exhibited significantly less ankle plantarflexion (*F*_1,24_ = 8.407, *p* = 0.008, *η*_p_^2^ = 0.259 large effect, Table 1) and less peak hip joint power (*F*_1,24_ = 7.416, *p* = 0.012, *η*_p_^2^ = 0.244 large effect, Table 2) than the normal arched group at CMJ take off. Additionally, the main effect of foot orthosis indicated that basketball players wearing foot orthosis demonstrated significantly less ankle eversion (*F*_1,24_ = 6.702, *p* = 0.016, *η*_p_^2^ = 0.218 large effect, Table 1) at CMJ take off than that of wearing control insole condition.

### Standing broad jump

There were no significant interactions between foot type and foot orthoses jump distance, kinematics and kinetic variables (*p* > 0.05, Tables 3 and 4). There were also no main effects of foot type (*F*_1,24_ = 0.197, *p* = 0.661, *η*_p_^2^ = 0.008 small effect) and foot orthosis (*F*_1,24_ = 2.661, *p* = 0.116, *η*_p_^2^ = 0.100 medium effect) on jump distance.

**Table 3:** Standing broad jump kinematics of participants when wearing control and prefabricated orthosis

Variables	Foot type group	Control	Orthosis	MD_orthosis_ [95% CI]	ANOVA *p*-value		
		(Mean ± SD)	(Mean ± SD)		Foot type	Orthosis	Interaction
Joint angles at take-off							
Hip - sagittal (°)	Normal arched	5.1 ± 6.9	5.8 ± 8.4	− 0.7 [− 2.9,1.5]	.945	.409	.799
	Flat-footed	5.1 ± 7.2	5.5 ± 7.1	− 0.0 [− 0.0,0.0]			
	MD_foot_ [95% CI]	0.0 [−5.7,5.78]	0.4 [−6.1,6.9]				
Knee - sagittal (°)	Normal arched	26.6 ± 11.2	26.1 ± 14.7	−0.5 [−4.3,3.2]	.803	.932	.723
	Flat-footed	24.9 ± 14.8	25.2 ± 14.4	0.3 [−2.4,3.0]			
	MD_foot_ [95% CI]	−1.8 [− 12.3,8.8]	−0.9 [− 12.9,11.0]				
Ankle - sagittal (°)	Normal arched	30.7 ± 5.6	30.2 ± 5.7	−0.2 [−1.7,1.5]	.050	.768	.989
	Flat-footed	25.6 ± 6.6	25.5 ± 5.9	−0.2 [−1.5,1.2]			
	MD_foot_ [95% CI]	−4.7 [−9.7,0.2]	− 4.7 [−9.5,-0.0]				
Ankle - frontal (°)	Normal arched	4.7 ± 5.9	3.8 ± 5.0	0.9 {−0.3,2.1]	.098	**.028**	.583
	Flat-footed	8.7 ± 5.0	7.2 ± 6.5	1.4 [−0.5,1.6]			
	MD_foot_ [95% CI]	−4.0 [−8.5,0.4]	−3.5 [−8.1,1.2]				
Peak angular velocity							
Hip - sagittal (°/s)	Normal arched	769.2 ± 266.6	692.2 ± 248.9	−77.0 [−282.6128.6]	**.013**	.420	.600
	Flat-footed	572.6 ± 138.4	556.2 ± 108.5	−16.4 [−61.2,28.4]			
	MD_foot_ [95% CI]	− 196.6 [− 378.8,14.5]	− 136.1 [− 302.0, 29.9]				
Knee - sagittal (°/s)	Normal arched	809.4 ± 269.7	787.0 ± 246.6	22.4 [−44.3,89.1]	.892	.457	.783
	Flat-footed	816.6 ± 249.0	806.3 ± 228.1	10.3 [−50.4,71.1]			
	MD_foot_ [95% CI]	−7.2 [− 221.3, 206.8]	−19.3 [− 215.2176.6]				
Ankle - sagittal (°/s)	Normal arched	1041.1 ± 120.4	1065.5 ± 205.0	24.4[−46.3,95.1]	.701	.909	.281
	Flat-footed	1085.0 ± 121.8	1065.2 ± 115.7	−19.8 [−46.8.7.3]			
	MD_foot_ [95% CI]	43.8 [−55.3142.9]	−0.3 [−142.5141.8]				
Ankle - frontal (°/s)	Normal arched	176.6 ± 103.8	247.0 ± 275.3	−70.4 [210.3,69.5]	.742	.614	.213
	Flat-footed	206.9 ± 120.2	177.0 ± 145.7	30.2 [−15.8,76.3]			
	MD_foot_ [95% CI]	−30.3 [−121.2,60.5]	70.3 [−118.4259.0]				

*MD*_*foot*_ mean difference in foot type (flat foot – normal arch), *MD*_*foot*_ mean difference in orthosis condition (orthosis – control), *CI* confidence intervals, *ANOVA* Analysis of Variance. *Significant differences (*p* < .05) are shown in bold

**Table 4:** Standing broad jump kinetics of participants when wearing control and prefabricated orthosis

Variables	Foot type group	Control	Orthosis	MD_orthosis_ [95% CI]	ANOVA *p*-value		
		(Mean ± SD)	(Mean ± SD)		Foot type	Orthosis	Interaction
Peak horizontal GRF (%BW)	Normal arched	0.49 ± 0.06	0.48 ± 0.66	−0.01 [−0.02,-0.00]	**.026**	**.001**	.623
	Flat-footed	0.44 ± 0.05	0.42 ± 0.05	−0.02 [− 0.03,0.00]			
	MD_foot_ [95% CI]	−0.05 [− 0.09,-0.01]	−0.06 [− 0.10,-0.01]				
Peak joint moment							
Hip - sagittal (Nm/kg)	Normal arched	5.99 ± 9.85	7.21 ± 21.43	−1.22 [−9.25,6.82]	.501	.661	.380
	Flat-footed	5.15 ± 11.07	1.53 ± 3.84	3.62 [−4.65,11.90]			
	MD_foot_ [95% CI]	0.84 [−7.66,9.34]	5.67 [−7.89,19.24]				
Knee - sagittal (Nm/kg)	Normal arched	0.97 ± 0.41	2.65 ± 6.94	−1.68 [−5.52,2.16]	.389	.414	.416
	Flat-footed	0.86 ± 0.19	0.88 ± 0.16	−0.02 [−0.07,0.03]			
	MD_foot_ [95% CI]	0.11 [−0.16,0.39]	1.78 [−2.57,6.12]				
Ankle - sagittal (Nm/kg)	Normal arched	1.01 ± 0.09	1.00 ± 0.12	−0.01 [− 0.04,0.02]	.649	.739	.488
	Flat-footed	1.02 ± 0.09	1.03 ± 0.11	0.00 [−0.03,0.04]			
	MD_foot_ [95% CI]	0.01 [−0.06,0.08]	0.03 [−0.07,0.12]				
Ankle - frontal (Nm/kg)	Normal arched	0.09 ± 0.07	0.08 ± 0.06	0.01 [0.01,0.03]	.111	**.036**	.511
	Flat-footed	0.14 ± 0.07	0.11 ± 0.06	0.02 [−0.00,0.05]			
	MD_foot_ [95% CI]	−0.05 [− 0.10,0.01]	−0.04 [− 0.08,0.01]				
Peak joint power							
Hip - sagittal (W/kg)	Normal arched	116.97 ± 345.66	78.28 ± 261.29	38.69 [−11.78,89.17]	.567	.057	.606
	Flat-footed	75.92 ± 171.18	9.81 ± 18.77	66.10 [−50.69,182.89]			
	MD_foot_ [95% CI]	41.05 [− 193.42,275.53]	68.46 [−95.34,232.26]				
Knee - sagittal (W/kg)	Normal arched	8.67 ± 7.21	7.58 ± 2.69	−6.71 [−21.7,8.03]	.389	.414	.413
	Flat-footed	5.99 ± 1.99	5.95 ± 1.64	0.05 [−0.61,0.70]			
	MD_foot_ [95% CI]	2.67 [−1.96,7.31]	69.89 [−98.79,238.57]				
Ankle - sagittal (W/kg)	Normal arched	7.95 ± 1.89	8.28 ± 2.48	−0.33 [−1.19,0.53]	.511	.638	.419
	Flat-footed	8.67 ± 1.71	8.58 ± 1.68	0.08 [−0.38,0.56]			
	MD_foot_ [95% CI]	−0.71 [−2.20,0.77]	− 0.30 [− 2.09,1.49]				
Ankle - frontal (W/kg)	Normal arched	0.21 ± 0.23	0.18 ± 0.14	0.03 [−0.10,0.16]	.188	.581	.807
	Flat-footed	0.13 ± 0.08	0.12 ± 0.15	0.01 [−0.04,0.07]			
	MD_foot_ [95% CI]	−0.08 [− 0.06,0.23]	−0.63 [− 0.05,0.18]				

*GRF* ground reaction force, *MD*_*foot*_ mean difference in foot type (flat foot – normal arch), *MD*_*foot*_ mean difference in orthosis condition (orthosis – control), *CI* confidence intervals, *ANOVA* Analysis of Variance. *Significant differences (*p* < .05) are shown in bold

The main effect of foot type indicated the flat-footed group produced lower hip angular velocity (*F*_1,24_ = 7.115, *p* = 0.013, *η*_p_^2^ = 0.229 large effect, Table 3) and lower peak horizontal GRF (*F*_1,24_ = 5.594, *p* = 0.026, *η*_p_^2^ = 0.189 large effect, Table 4) at SBJ take off than the normal arched group. Additionally, there was a statistically significant effect of foot orthosis on ankle eversion (*F*_1,24_ = 5.453, *p* = 0.028, *η*_p_^2^ = 0.185 large effect) (Table 3), peak horizontal GRF (*F*_1,24_ = 13.672, *p* = 0.001, *η*_p_^2^ = 0.363 large effect) and peak ankle frontal moment (*F*_1,24_ = 4.932, *p* = 0.036, *η*_p_^2^ = 0.170 large effect) at SBJ take off (Table 4), indicating that foot orthosis conditions was significantly less ankle eversion, lower peak horizontal GRF, and peak ankle frontal moment (*p* > 0.05, Tables 3 and 4).

## Discussion

Our study aimed to investigate the effects of foot type and foot orthosis on the jumping performance and lower limb biomechanics in trained athletes. Our main results were 1) There was no difference in jump height or jump distance regardless of foot types and foot orthoses. 2) In CMJ, the flat-footed group displayed less plantarflexion and peak hip joint power than normal-arched group; foot orthoses reduced ankle eversion at take-off compared to flat neutral insoles. 3) In SBJ, the flat-footed group produced less peak horizontal GRF and lower hip angular velocity than the normal-arched group; foot orthoses reduced ankle eversion, peak horizontal GRF and ankle frontal plane moment at take-off compared to flat neutral insoles.

### Effect of foot type

During CMJ take-off, there was less ankle plantarflexion at the instant of take-off in the flat-footed group compared to the normal-arched group. This was consistent with the findings of Fu and his colleagues [2]. During the push-off phase of the jump, the gastrocnemius/soleus complex contracts concentrically and, through the taut Achilles tendon, acts as a spring to propel the body [32]. Greater plantarflexion angles were detected in the normal arched group compared to the flat-footed group and therefore, better jump heights and distances would have been expected in the normal arched group. However, in our study, there was no difference in jump performance between the groups even though the normal-arched group pushed off with greater plantarflexion angles. The gastrocnemius/soleus complex has been found to stretch and recoil in a catapult-like fashion, maximising ankle power significantly [33]. While greater plantarflexion at take-off might infer a better spring mechanism, the power generated by the elastic recoil is first dependant on the initial stretch. Therefore, future studies may need to investigate the ankle dorsiflexion angle just prior to take-off in order to confirm which group had a more efficient spring mechanism at the ankle.

Interestingly, individuals with flatter feet have been reported to have greater ankle muscle strength [34]. There may be a possibility that the increased ankle strength of the flat-footed group had compensated for the lack of the overall advantage provided by the spring mechanism of the ankle, resulting in similar jump performances. On the other hand, one may argue that flat-footed individuals would require greater effort (i.e., ankle strength) to maintain a similar level of performance as their normal- arched counterparts. This suggests that the flat-footed individuals may tire out faster [35] resulting in inferior jump performance sooner in the game [36]. Studying performance stability and biomechanics associated with foot-type seems helpful to explain the movement control strategies across different intensity/duration of movements.

For joint kinetics, the flat-footed group also jumped with significantly less hip joint power during CMJ take-off than the normal-arched group, but there were no differences in peak knee and ankle powers. Higher knee and ankle joint powers have been associated with better CMJ jump performance [37]. It would seem that the reduction in hip joint power does not influence jump performance and could partly explain the lack of difference in jump distances despite lower hip power found amongst flat-footed basketball players.

Our results showed that the flat-footed group produced lower horizontal GRF in SBJ than the normal-arched group, inferring that they may not be able to push off as efficiently. In our study, we found that there was a significantly lower hip angular velocity in the flat-footed group compared to the normal-arched group, but there was no corresponding reduction in hip power generated. This may explain the similar jump performance found in our study. It would seem that flat-footed individuals can perform just as well as their normal arched counterparts. The differences in biomechanics of the lower extremity may indicate that flat-footed individuals have to work harder to achieve the same performance and may infer that flat-footed individuals may experience fatigue earlier than their normal arched counterparts. Foot type accounted for 25.9% variance in ankle plantarflexion, 24.4% variance in hip joint power and 22.9% variance in hip angular velocity (large effect) at CMJ while accounted for 18.9% variance in peak horizontal GRF (small effect) at SBJ. Further studies need to be done to investigate the impact of these biomechanical changes on their clinical implications such as muscle use.

### Effect of foot orthosis

As foot orthoses are hypothesised to provide medial arch support and reduce rearfoot eversion [8, 18]. Although pronation is a tri-planar motion, involving inversion/eversion, dorsiflexion/plantarflexion and adduction/abduction, it is expected that due to the medial support, kinematic differences would be seen primarily in the frontal plane. Our results in CMJ and SBJ support the contention that when prefabricated orthoses were used, the ankle was in a more inverted position during take-off. In previous studies on walking and running, foot orthoses have been reported to produce 2° less eversion during heel raise in fast walking [38] and 3° less eversion during running [39]. Our findings showed that the use of foot orthoses resulted in a reduction between 1 and 3° of ankle eversion for all participants during CMJ and SBJ. These small magnitudes of change in ankle frontal plane angles found in our study are consistent with other studies measuring the effects of foot orthoses on foot kinematics [38, 39]. Our results show that an increase in ankle stability would not relate to jumping performance and is consistent with another study that increased ankle stability using collar height and heel counter stiffness of footwear [30]. One possible explanation is that when jumping from a stationary position, the foot pushes off using mainly the forefoot [3]. This is unlike other forms of locomotion such as walking and running where there is considerable rearfoot and midfoot involvement during ground contacts. The foot orthoses used in our study provide support primarily in the rearfoot and midfoot areas [22], which may have caused changes in the shape and biomechanics on these regions of the foot. The forefoot, which is used more during jumps, may not be affected by the prefabricated foot orthoses used in our study and therefore may not be effective in enhancing jumping performances. Other jumping studies on basketball footwear have shown that changing the forefoot structures (e.g., bending stiffness) of a shoe can play an important role in jumping performance [25, 28]. Future studies may consider if a forefoot varus wedge and segmented stiffness insoles may improve jumping performances in basketball players. Foot orthosis accounted for large effect sizes in ankle eversion (21.8% variance) in CMJ and accounted for large effect in ankle eversion (18.5% variance) and peak ankle frontal moment (17% variance) at SBJ. Although the magnitude of change was small, the large effect sizes found in this study would encourage future longitudinal studies to ascertain the impact of these biomechanical differences to potentially improve movement analyses to tailor training strategies to improve jump performances.

### Limitations

There were a few limitations in our study. The prefabricated insoles were not customised to the individual athletes’ foot type and requirements. The contour of the orthoses may affect fit and comfort of the individuals and thus influence performances. Future studies may be required to compare the efficacy of prefabricated and custom-made orthoses on jump performance. Furthermore, our participants had only 2 min to get used to jumping using the supplied prefabricated orthoses. Future studies may consider providing a sufficiently long wear-in period before trials commenced. Since the forefoot is utilised primarily during jumps, the assessment of forefoot function, including factors such as first metatarsophalangeal joint mobility [40] should be considered in future studies that assess jump performance and biomechanics. This will provide a more holistic reflection of foot function during jumps. Finally, we statistically compared a large number of kinetic and kinematic variables to provide a comprehensive analysis of the jumping biomechanics. There is a possibility of TypeI errors when interpreting findings as the alpha value has not been adjusted.

## Conclusion

Similar vertical and horizontal jump performances were observed between flat-footed and normal-arched basketball players, regardless of whether foot orthoses were used. Compared to normal-arched athletes, flat-footed players exhibited less ankle plantarflexion and peak hip joint power in CMJ, and lower horizontal GRF and lower hip angular velocity in SBJ during the take-off phase. The use of foot orthoses reduced ankle eversion in both jump tasks, and reduced horizontal GRF and ankle moment in SBJ during the take-off phase. Since these kinematic and kinetic differences did not affect jump performances, it is recommended that basketball coaches and players should not view flat foot as a disadvantage in terms of jumping capability. Regardless of foot type, there was insufficient evidence found in our study to support the use of foot orthoses for improving countermovement and standing broad jump performances in trained basketball players.

## Abbreviations

ANOVA: Analysis of variance
BW: Body weight
CI: Confidence interval
CMJ: Countermovement jump
GRF: Ground reaction force
MD: Mean difference
NCSP: Neutral calcaneal stance position
SBJ: Standing broad jump
